# Tear evaluation by anterior segment OCT in dry eye disease

**DOI:** 10.22336/rjo.2021.6

**Published:** 2021

**Authors:** Diana Mădălina Popovici, Ana Banc

**Affiliations:** * ENT Department, County Emergency Hospital Cluj-Napoca, Romania Department of Ophthalmology, “Iuliu Hațieganu” University of Medicine and Pharmacy Cluj-Napoca, Romania

**Keywords:** dry eye disease, tear film thickness, tear meniscus area, tear meniscus height

## Abstract

**Objective**.

Dry Eye Disease (DED) is a multifactorial disorder, centered by loss of tear homeostasis. The diagnosis represents a challenge in the absence of a gold standard, so different questionnaires and techniques are combined. Considering that a low lacrimal secretion and a high rate of evaporation can determine changes in the tear film, the question that arises is if there are differences between the tear film thickness and the tear meniscus values of patients with DED compared to healthy volunteers, and if so, if they can be proposed as an objective diagnosis technique using Optical Coherence Tomography (OCT).

**Materials and methods**.

Ocular Surface Disease Index (OSDI) was used together with examiner confirmation for the diagnosis of DED. All the images were acquired using anterior segment Spectral Domain - OCT. Measurements were calculated using ImageJ. IBM SPSS Statistics was used for data analysis. Statistical significance was achieved if p value was <0.05, with 95% confidence intervals.

**Results**.

There were no statistically significant differences between the two groups concerning tear film thickness for the right or left eye (p=0.895 and p=0.178, respectively, p >0.05) or the difference between them (p=0.858, p >0.05). Tear meniscus area and height for each eye and the difference between the eyes reported no significant difference between the healthy and the DED volunteers.

**Conclusion**.

Tear film thickness does not record statistically significant differences between the DED and the healthy group, and neither does the sagittal area, the tear film height, or the difference between them when acquired with OCT.

**Abbreviations:** DED = dry eye disease, ASOCT = anterior segment optical coherence tomography, REFT = right eye tear film thickness, LEFT = left eye tear film thickness, DifFT = difference between the two eyes for tear film thickness, RETMA = right eye tear meniscus area, LETMA = left eye tear meniscus area, DifTMA = difference between the two eyes for tear meniscus area, RETMH = right eye tear meniscus height, LETMH = left eye tear meniscus height, DifTMH = difference between the two eyes for tear meniscus height

## Introduction

The tear film plays a crucial role in lubrication of the ocular surface and in foreign bodies removal [**1**]. It is divided into two compartments, the preconjunctival and the precorneal compartment. Thickness of the tear film is only known for the precorneal segment, the medium values being approximately 3 µm [**2**]. The following factors can influence the lacrimal film: hydration status, lacrimal lipidic layer characteristics, palpebral aperture dimensions, the interval between two blinks, tear film stability and environmental conditions [**3**]. The precorneal lacrimal film is subdivided into two layers: the superficial one is lipidic, secreted by the Meibomian glands, and the deep layer is muco-aqueous. The lipid layer spreads over the muco-aqueous one with every blink, contributing to the tear film stability. Lipidic deficiency slows down the layer’s distribution [**4**], contributing to the tear film instability. If the muco-aqueous layer becomes thinner, it can determine an aqueous-deficient dry eye. Dry eye disease (DED) is a multifactorial disorder, centered by loss of homeostasis of the tear film [**5**]. Despite a difficult comparison between the studies due to their heterogeneity, the prevalence of symptomatic disease increases in female patients only with age, evident differences occurring only after 50 years old. The prevalence of the disease is also higher in Asian patients when compared to Caucasian ones [**6**]. Incidence in symptomatic DED patients is poorly studied, but it has been reported in one study as 13.3% in 5 years (Confidence interval 95%, [12.0-14.7%]) in a Caucasian population with ages between 48 and 91 years [**7**], and 21.6% in 10 years (Confidence interval 95%, [19.9-23.3%]) in a population with ages between 43 and 86 years [**8**]. Quality of life is negatively influenced by the disease [**9**], since it can alter daily activities like reading, driving and work-related activities.

Hyperosmolarity and tear film instability are two important etiological factors involved in the lacrimal film homeostasis. Thus, the lacrimal film and its changes require a careful examination. The absence of a gold standard for the diagnosis of DED represents, currently, an obstacle in the way of defining on one hand, and facile identification and treatment of the syndrome, on the other hand. At present, a combination of questionnaires and maneuvers are used for the diagnosis. Even though the relationship between DED signs and symptoms is not linear and is dependent on the type of DED involved, quantification of symptoms is important as a screening method that can signal the possibility of DED and the need for further examination, but is also necessary for disease progression and treatment response follow-up. Recommendations are that a symptoms’ questionnaire is administered during the first visit [**10**]. Since low tear film secretion, as well as a high rate of evaporation, could determine changes in the tear film dimensions, the question that arises is if there are differences between the thickness of the tear film and the tear meniscus dimensions of healthy volunteers and DED patients. If there are recorded differences, are they enough to represent an objective diagnosis method? Optical Coherence Tomography (OCT) is a tool used for assessing different anterior segment eye variables, having the advantage of being non-invasive, besides the fact that it does not determine reflex lacrimation, as does fluorescein instillation, video or reflexive meniscometry. Moreover, a difference in osmolarity higher than 8 mOsm/ L between the two eyes is considered significant for the diagnosis of DED [**11**]. Since hyperosmolarity stimulates evaporation and a certain value of the difference between the eyes has been proposed as means of diagnosis for DED, another question we asked is if the difference between the eyes of the tear variables of DED patients differs from that of healthy volunteers, and therefore, can be used as a diagnosis tool.

The aim of the paper was to offer an answer to the questions asked above, evaluating the values obtained from a group of healthy individuals compared to the ones obtained from a group of DED patients. This study applied the OSDI (Ocular Surface Disease Index) to quantify the volunteers’ symptomatology, in association with an ophthalmologic examination, to confirm the diagnosis of DED, followed by tear film and tear meniscus measurements using a rapid and non-invasive method, anterior segment OCT (ASOCT).

## Materials and methods

The current study was approved by “Iuliu Hațieganu” University of Medicine and Pharmacy Ethics Committee, Cluj-Napoca, Romania. The procedures were explained to all the volunteers and an informed consent of participation was obtained in all the cases. The criteria of inclusion in the study were the access to internet and the availability of attending both phases of the study. The criteria of exclusion from the study were represented by the regular use of artificial tear drops or other topic ophthalmic products and other diseases of the surface of the eye.

The materials we employed were OSDI (Romanian translation), as recommended by the TFOS DEWS II Diagnostic Methodology report [**10**], in the form of an auto-administered online questionnaire, because literature data showed there were no differences between hetero- or auto-administration [**12**]. OSDI evaluated the frequency of symptoms, environment stimuli, and quality of life, when referring to sight. It included six questions about sight deficiency (blurred vision, weak sight) and visual function (difficulty while reading, driving at night, working on a computer, watching TV). Based on the result, the person had DED or not. For confirmation of DED diagnosis, the volunteers were examined at the biomicroscope. Anterior segment image acquisition was performed using Spectral Domain-ASOCT (Heidelberg Engineering, Heidelberg, Germania), with predefined scanning protocol “Scan 8”. Confirmation and image acquisition were both conducted at the Ophthalmology Clinic in Cluj-Napoca, Romania.

**Fig. 1:**
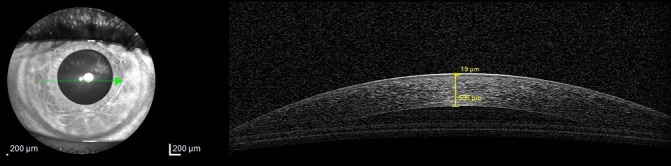
Tear film thickness measurement technique. From surface to depth, two values can be seen in every image: the first, superficial one, is of the tear film thickness, while the second one is of the corneal thickness

**Fig. 2:**
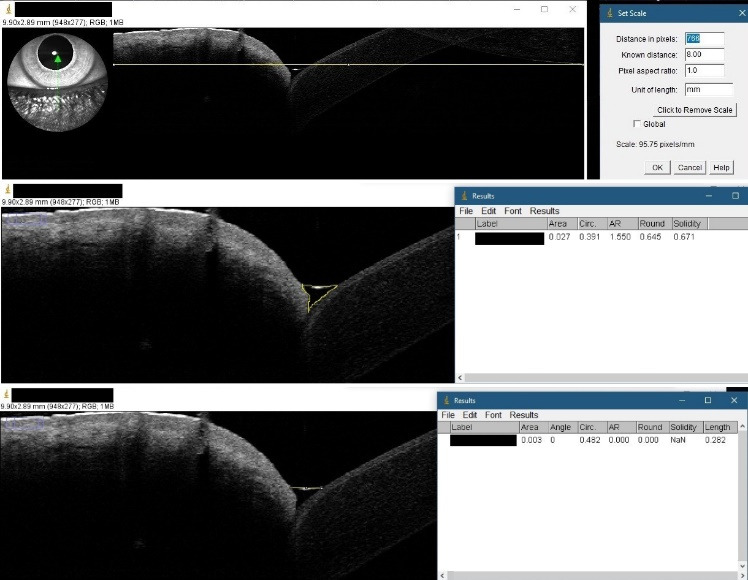
Example of values assessment using ImageJ software. **a.** Scale setting. **b.** Tear meniscus area measurement (TMA). **c.** Tear meniscus height measurement (TMH)

The first phase was the online questionnaire administration. After each volunteer submitted the answer, the score was calculated for everyone and communicated via an e-mail, in which information about the next phase was detailed. Two groups were formed: the DED group and the healthy volunteers group. A person had DED if the score was 13 or higher and the ophthalmologic examination was positive for the signs of DED. The following images were obtained using OCT, bilaterally: a transversal section through the center of the pupil for the lacrimal film thickness measurements and a vertical, central section of the inferior tear meniscus for area and height evaluation. Measurements were performed under the same environmental conditions. Tear film thickness was measured using the OCT’s software (**Fig. 1**). For area and height of the inferior tear meniscus calculation, ImageJ, 1.52a version (National Institutes of Health and the Laboratory for Optical and Computational Instrumentation, University of Wisconsin, WI, USA) was employed (**Fig. 2**). The length of the section (8 mm) was used as scale. Uninterpretable acquisitions were excluded. The difference between the two eyes of each participant were also determined.

Statistical analysis was performed using IBM SPSS Statistics 1.0.0.1174 version, 64-bit edition (IBM, Armonk, NY, USA). The following variables were defined: presence or absence of DED, lacrimal film thickness, lacrimal meniscus area and height for right and left eye and the differences between the eyes for the last three variables. The variable distribution was checked using Shapiro-Wilk test and if they had a normal distribution, Student t test for independent samples was applied, if not, non-parametric tests for independent samples were employed (Mann-Whitney U). The interpretation of results was performed according to p value, statistical significance being achieved when p<0.05, with 95% confidence intervals.

## Results

Patient demographics are presented in **Table 1**.

The first phase recorded 55 answers to OSDI questionnaire, of which 42 presented for the second step, therefore only their data were included in the study. The control group was made up of 15 individuals (36%), while the case group included 27 people (64%). The cases were subdivided into mild (n = 10), medium (n = 8) and severe (n = 9) (**Table 1**).

**Tabel 1:** Patient demographics, tear film and tear meniscus variables

Age, y (median, min-max)	25, 20-63
Female (n, %)	22 (52,38%)
Male (n, %)	20 (47,62%)
Control group (n, %)	15 (36%)
DED patients (n, %)	27 (64%)
Mild (n, %)	10 (37%)
Medium (n, %)	8 (30%)
Severe (n, %)	9 (33%)
Lacrimal variables	
REFT, µm (median, min-max)	18.71 (10-25)
LEFT, µm (median, min-max)	18.59 (12-28)
DifFT, µm (median, min-max)	3.75 (0-10)
RETMA, mm2 (median, min-max)	0.046 (0.008-0.340)
LETMA, mm2 (median, min-max)	0.051 (0.007-0.437)
DifTMA, mm2 (median, min-max)	0.042 (0.000-0.397)
RETMH, mm (median, min-max)	0.380 (0.157-1.126)
LETMH, mm (median, min-max)	0.378 (0.160-1.450)
DifTMH, mm (median, min-max)	0.169 (0.000-1.004)
y = years, n = number, min = minimum, max = maximum, REFT = right eye tear film thickness, LEFT = left eye tear film thickness, DifFT = difference between right and left eye tear film thickness, RETMA = right eye tear meniscus area, LETMA = left eye tear meniscus area, DifTMA = difference between right and left eye tear meniscus area, RETMH = right eye tear meniscus height, LETMH = left eye tear meniscus height, DifTMH = difference between right and left eye tear meniscus height	

The normality test indicated that the right eye tear film thickness (REFT), left eye film thickness (LEFT) and the difference between the eyes (DifFT) followed a normal distribution, while the right and left eye tear meniscus height (RETMH and LETMH, respectively), and area (RETMA and LETMA, respectively) and the difference between the eyes (DifTMH and DifTMA) did not respect a normal distribution. Levene’s test for equality of variances was applied for REFT, LEFT and DifFT and equal variances were assumed (p > 0.05), thus the Student t test was utilized. Neither REFT, nor LEFT presented statistically significant differences between the two groups (pREFT = 0.895, pLEFT = 0.178, p > 0.05). The difference in thickness between the two eyes did not vary in the two samples (pDifFT = 0.858, p > 0.05). Mann-Whitney U test was employed for each eye’s TMH, TMA and for the difference in height and area between the two eyes, and p value was above 0.05 in all cases. The results are summarized in **Table 2**.

**Tabel 2:** p values for tear film and tear meniscus variables

Variable	p value	Confidence interval*	
		Lower	Upper
REFT	0.895	-2.082	2.375
LEFT	0.178	-3,813	0,733
DifFT	0.858	-1,687	2,016
RETMA	0.319		
LETMA	0.189		
DifTMA	0.388		
RETMH	0.247		
LETMH	0.490		
DifTMH	0.372		
REFT = right eye tear film thickness, LEFT = left eye tear film thickness, DifFT = difference between right and left eye tear film thickness, RETMA = right eye tear meniscus area, LETMA = left eye tear meniscus area, DifTMA = difference between right and left eye tear meniscus area, RETMH = right eye tear meniscus height, LETMH = left eye tear meniscus height, DifTMH = difference between right and left eye tear meniscus height. *Confidence interval is given for the variables with normal distribution.			

## Discussion

The results we obtained indicated that there were no significant differences recorded among the DED group and the control group when referring to the film thickness, the tear meniscus height and area, measured with ASOCT. Therefore, even though hyperosmolarity intensified tear film evaporation and promoted its instability, the tear film and the tear meniscus value did not report significant changes. One explanation could be that in the incipient stages of the disease, only points of hyperosmolarity appeared, and the tear thickness remained unaltered. On the other hand, irritation of the free corneal nerve endings, determined by hyperosmolarity, stimulated lacrimal reflex secretion, thus, the quantity of the tear film could remain relatively constant for some individuals, for a certain period [**13**].

The literature in this field shows a difference between the DED and the control groups using Fourier Domain-OCT (FDOCT), but the existing papers present certain variations from the current paper, which were detailed below. There is only one study measuring tear film thickness, while for tear meniscus, there are several studies published.

A study conducted by Qui X et al. [**14**], identified differences between the two groups, but the inclusion criteria were the presence of symptoms and modified values of Schirmer test and TBUT (tear break-up time). In the research, the variables of tear meniscus correlated with the Schirmer test, TBUT, but not with corneal staining or DEQ (dry-eye questionnaire). Another study directed by Nguyen P et al. [**15**], studied the correlation between tear meniscus measurements and Indiana Dry Eye Questionnaire 2002, TBUT, rose Bengal staining, Schirmer test. The values obtained for tear meniscus were positively correlated only with Schirmer test. An important mention is that the examined group consisted only of patients, all under treatment with tear drops and two thirds under treatment with ciclosporin, thus already diagnosed with DED and under treatment with step 1 or 2 medication [**16**], which can influence the signs and symptoms of DED. If Schirmer test is used as the main criteria of DED diagnosis, as in *Optical Coherence Tomography for Measuring the Tear Film Meniscus: Correlation with Schirmer Test and Tear-Film Breakup Time* [**17**], TBUT, TMH and TMA record statistically significant different values in DED patients. Another study [**18**] compared the superior and inferior tear meniscus variables and tear film thickness of DED patients and healthy volunteers. The diagnosis method was a combination of McMonnies questionnaire, Schirmer test and slit-lamp examination. No differences in tear film thickness and superior tear meniscus were observed, but for inferior tear meniscus area and height lower values were obtained in DED patients. The technique used for measurements was different than in the present paper. The last research used fluorescein staining, BUT (break-up tear time) and Schirmer test and/ or symptoms as diagnostic criteria [**19**]. Every study listed used Schirmer test as a diagnosis tool, but the Schirmer test without anaesthetic drops is a diagnostic test recommended only for confirmed severe aqueous deficiency. The test is variable and invasive and, in patients with evaporative disease, the insertion of the strip could lead to reflex tearing response, therefore masking a discrete reduction in tear volume [**10**]. TFOS DEWS II Diagnosis Methodology report [**10**], the latest guideline for DED diagnosis and treatment, does not recommend it as a routine diagnostic test of tear volume.

Limitations to our study consisted of a possible variation of the moment of image acquisition from the last blink, that can influence the tear film thickness and tear meniscus variables and age distribution. Standardization of tear variables acquisition using ASOCT could lead to more reliable and reproductible results. Moreover, a larger patients’ sample and pairing healthy volunteers with DED patients according to their age and sex could eliminate the errors rising from differences in variables.

To our knowledge, this is the first study to examine tear variables without using Schirmer test as a diagnosis tool, only OSDI and examiner confirmation, followed by ASOCT examination. There are no statistically significant differences between the DED patients and the healthy volunteers through the diagnosis method we used, so a sensitive, non-invasive, rapid diagnosis tool remains a goal. Another novelty brought by this study was the examination of both eyes, because there are differences recorded between the eyes. Furthermore, the hypothesis that there could be differences between values of tear variables of the eyes, and they could serve as diagnosis criteria, in the same manner hyperosmolarity differences between the eyes can be used to confirm a DED diagnosis, was, to our knowledge, for the first time formulated in this study. Further studies are required to standardize the tear variables measurements through OCT, the acquisition timing, and larger, paired groups should be included. DED can influence the quality of life, and, thus, have a strong impact on patients, so an early diagnosis and treatment is not only desirable, but important for the management of the disease and the well-being of the patient.

## Conclusion

Tear film and tear meniscus variables do not record differences between healthy volunteers and DED patients when measured with ASOCT. Standardization of image acquisition using ASOCT and larger, paired groups could lead to more refined results while a feasible, non-invasive diagnosis tool for DED remains a desiderate yet to be attained.

**Conflict of Interest**

Authors state no conflict of interest.

**Informed Consent and Human and Animal Rights statements**

An informed consent has been obtained from all individuals included in this study.

**Authorization for the use of human subjects**

Ethical approval: The research related to human use complies with all the relevant national regulations, institutional policies, is in accordance with the tenets of the Helsinki Declaration, and has been approved by “Iuliu Hațieganu” University of Medicine and Pharmacy Ethics Committee, Cluj-Napoca, Romania.

**Acknowledgements**

None.

**Sources of Funding**

None.

**Disclosures**

None.
